# Gemcitabine and carboplatin in carcinoma of unknown primary site: a phase 2 Adelaide Cancer Trials and Education Collaborative study

**DOI:** 10.1038/sj.bjc.6603440

**Published:** 2006-10-31

**Authors:** K B Pittman, I N Olver, B Koczwara, D Kotasek, W K Patterson, D M Keefe, C S Karapetis, F X Parnis, S Moldovan, S J Yeend, T J Price

**Affiliations:** 1 Department of Oncology, The Queen Elizabeth Hospital, Woodville Road, Woodville, South Australia 5011, Australia; 2 Cancer Centre, Royal Adelaide Hospital, Adelaide, South Australia 5000, Australia; 3 Department of Medical Oncology, Flinders Medical Centre, Bedford Park, South Australia 5042, Australia; 4 Cancer Centre, Ashford Hospital, Ashford, South Australia 5032, Australia

**Keywords:** carboplatin, carcinoma of unknown primary, elderly, gemcitabine

## Abstract

Cancer of unknown primary site (CUP) represents up to 5% of all cancer diagnoses and is associated with poor survival. We have performed a prospective multicentre phase 2 trial to evaluate efficacy and toxicity of the combination of gemcitabine (G) and carboplatin (C) for patients with CUP. Patients with histologically confirmed metastatic carcinoma in which the primary site of cancer was not evident after prospectively designated investigation and who had ECOG performance status 0–2 were treated with G 1000 mg m^−2^ intravenously (i.v.) days 1 and 8, and C AUC 5 i.v. on day 8 every 3 weeks to a maximum of nine cycles. The primary end points were response rate, and toxicity, with secondary end points of progression-free survival and overall survival. Fifty-one (23 male, 27 female) patients were enrolled (one patient ineligible), with a median age of 69 years (range 41–83 years). Fifty patients were evaluable for toxicity and 46 patients were evaluable for efficacy. The overall response rate to the GC regimen was 30.5%. With a median follow-up of 24 months, the median progression-free survival was 18 weeks (4.2 months) and the median overall survival was 34 weeks (7.8 months). The frequency of grade 3 or 4 toxicity was low. Nausea/vomiting was the most common side effect, but was usually only mild in severity. Uncomplicated neutropenia (14%), thrombocytopenia (10%) and anaemia (8%) were the most common causes of grade 3–4 toxicity. The regimen was very well tolerated, particularly in the elderly. The GC regimen is an active regimen in CUP with excellent tolerability and should be considered particularly for elderly patients with CUP.

Cancer of unknown primary site (CUP) represents up to 5% of all new cancer diagnoses, of which 60% are adenocarcinomas. When CUP is diagnosed in the absence of specific symptoms to suggest a primary site of malignancy, there is a high chance that extensive evaluation will not identify a primary site (Greco and Hainsworth, 2001).

There are several clinical circumstances when specific subgroups of patients presenting with CUP may be treated with tumour-specific chemotherapy, with an expectation of significant efficacy. These include: (i) peritoneal carcinomatosis or malignant ascites in women, particularly with serous cancers where ovarian cancer protocols are often effective, (ii) women with axillary lymph node metastases where treatment for occult breast carcinoma has been shown to be effective, (iii) male patients with osteoblastic bone metastases where empiric hormonal manipulation for metastatic prostate cancer would seem worthwhile, (iv) young males with undifferentiated carcinomas, involving retroperitoneal mediastinal lymph nodes where empirical treatment for germ cell tumour may be appropriate, (v) patients who present with metastatic squamous cell carcinoma in high cervical nodes where radical treatment for head and neck cancer is usually undertaken (Lenzi *et al*, 1997).

However, about 90% of patients with CUP are not in the clinical subgroups previously outlined. Various combinations of chemotherapy have been shown to have some effect in these circumstances. Response rates range from 0 to 50% with an average response rate of about 20–30%. Some of the most active older combinations have contained doxorubicin or cisplatin (Sporn and Greenberg, 1993; Parnis *et al*, 2000). More recently, newer cytotoxics, such as taxanes, irinotecan and gemcitabine (G), have been used in combination chemotherapy with more consistent response rates in the order of 25–40%, although some of these combinations have been associated with significant toxicity (Greco *et al*, 2002; Culine *et al*, 2003; Park *et al*, 2004).

In most series, the median survival rate for patients treated for CUP is 6–8 months. As such, all treatment in this clinical setting should be considered palliative. A concern with many combinations, including those containing agents such as doxorubicin, cisplatin, etoposide, taxanes and irinotecan, is that they are likely to produce significant toxicity, such as nausea/vomiting, myelosuppression and alopecia. Despite the significant improvement in general community education about cancer and cytotoxic chemotherapy, the side effects of nausea/vomiting and alopecia remain high on the list of patient concerns when confronting chemotherapy treatment. Clearly, combinations with good efficacy but with better side-effect profile are warranted in the palliative setting of CUP.

Platinum-based doublets or triplets remain the most commonly reported protocols for CUP. Carboplatin (C) is often now used in numerous clinical situations where cisplatin was traditionally considered standard. Carboplatin produces more myelosuppression than cisplatin, but has the advantage of inducing less nausea, vomiting and neurotoxicity.

Gemcitabine as a single agent has been shown to have significant activity in small-cell and nonsmall-cell lung cancer, breast cancer, ovarian cancer, hormone refractory prostate cancer, pancreatic cancer, bladder cancer, lymphoma and head and neck cancer. Its major toxicity is myelosuppression, which tends to be dose dependant. Although gemcitabine is now approved and licensed in Australia for numerous malignancies, at the time this trial was commenced in 2001, it was only routinely available in Australia in the settings of lung cancer and pancreatic cancer.

The combination of GC has the advantage of being able to be administered rapidly in an outpatient setting with myelosuppression (usually dose dependent) being the only frequent major toxicity. Significant nausea, vomiting and alopecia are rare. The GC combination has now been reported in numerous clinical situations, including nonsmall-cell lung cancer, bladder cancer and ovarian cancer with good tolerance and significant activity (Langer *et al*, 1998; Linardou *et al*, 2004; Pfisterer *et al*, 2005; Sedeholm *et al*, 2005). The spectrum of activity of the GC combination is such that it would appear to be an ideal combination to use in the management of CUP, provided that colorectal cancer can be reasonably excluded.

We instigated a prospective open-label phase 2 trial to determine the efficacy and tolerability of the GC combination as first-line therapy in CUP patients presenting to cancer centres across metropolitan Adelaide in South Australia, Australia.

## PATIENTS AND METHODS

Patients who presented to cancer centres in Adelaide, South Australia with histologically confirmed metastatic carcinoma (proven by immunohistochemistry) for which no primary site of disease was found after standard evaluation were eligible for study. Only cases of adenocarcinoma, large-cell carcinoma, undifferentiated carcinoma and carcinoma ‘not otherwise specified’ were included. Squamous cell carcinoma, small-cell carcinoma/neuroendocrine carcinoma and serous/papillary carcinoma were not included. Prior chemotherapy for CUP was not permitted.

Routine evaluation included CT scan of chest, abdomen and pelvis, mammography in the female patient, CA125 in the female patient, and PSA, alpha feto-protein and betaHCG in the male patient. Additionally, designated investigation of the following scenarios was mandatory: upper GI endoscopy for a history of significant dysphagia or marked dyspepsia or intermittent epigastric pain; colonoscopy for a history of recent change in bowel symptoms, rectal bleeding or iron deficiency anaemia, or significantly elevated CEA level; bronchoscopy for a history of recent change in cough or wheeze or history of haemoptysis; and cystoscopy for a history of recent haematuria.

Patients had to be 18 years of age or over and had to have ECOG performance status of 0–2. Adequate haematological, hepatic and renal function as directed by protocol was mandatory. Patients had to have a life expectancy of greater than 3 months in the opinion of the treating clinician. Patients were required to give written informed consent before commencement of any specific studies and specific investigations and had to be willing and able to comply with the protocol for the duration of the study. Patients with either measurable or evaluable disease were eligible. The study was approved by the ethics committees of each of the treating institutions.

Patients who entered the trial underwent treatment with G 1000 mg m^−2^ as a 30 min infusion on days 1 and 8, with C administered in an AUC5 (calculated using the Calvert formula (Calvert *et al*, 1989)) over 1 h on day 8, with treatment repeated on a 3 weekly basis. The antiemetic regimen was left to the discretion of investigator/clinician.

During treatment, patients were reviewed on days 1 and 8 of each treatment cycle and underwent general physical examination, toxicity assessment and assessment of ECOG performance status. Haematological and biochemical indices were checked on days 1 and 8.

Formal tumour evaluation was performed after every third cycle of chemotherapy. Response evaluation was determined according to WHO criteria (Miller *et al*, 1981). Quality of life evaluation was performed at baseline and after every third cycle of chemotherapy.

Dose reductions were determined by protocol-defined observed toxicities and were based upon the starting dose. Dose escalations were not allowed and colony-stimulating factors (CSFs) were not permitted in this study. Patients with objectively defined tumour progression were withdrawn from the study. Patients could be withdrawn from study at discretion of investigator/clinician or at the request of the patient. Patients could continue with treatment regimen to a maximum of nine cycles if there was no evidence of tumour progression and good tolerability.

The total sample size required to demonstrate a response rate of 40%, assuming a historical response rate of 20%; was 50 patients, with 90% power, a significance level of 0.05, and two-sided significance testing (Fleming, 1982).

## RESULTS

Between June 2001 and January 2004, 51 patients were entered into the trial. One patient was excluded as the primary site of cancer (colon) was discovered during the first cycle of treatment. Fifty patients were therefore available for toxicity analysis. Forty-six patients were eligible for response analysis. Four patients could not be fully evaluated for response owing to early withdrawal or death within the first 3 weeks of therapy. Response was analysed according to both evaluable and ‘intention-to-treat’ (ITT) criteria. All patients were included in progression-free survival and overall survival analysis. Patient characteristics and treatment parameters are detailed in Table 1.

The mean relative dose intensity (RDI) achieved was 0.80 of the planned dose. The median RDI for patients receiving four cycles or more was 0.86 of the planned dose.

### Toxicity

Median number of cycles of chemotherapy was four (range 1–9). The side effects of the chemotherapy protocol were as described in Table 2.

Forty-eight per cent of patients experienced a grade 3 or greater toxicity. The majority of this was haematological toxicity: 14% neutropenia, 10% anaemia, and 8% thrombocytopenia. The febrile neutropenia rate was 6%. There was no alopecia and only one episode of grade 3 nausea. There was one death that may be partly attributed to neutropenic sepsis. This patient had significant pulmonary metastases and malignant pleural effusion and presented with respiratory failure, renal failure and febrile neutropenia. He died shortly after admission to hospital.

### Efficacy

**Figure 1:**
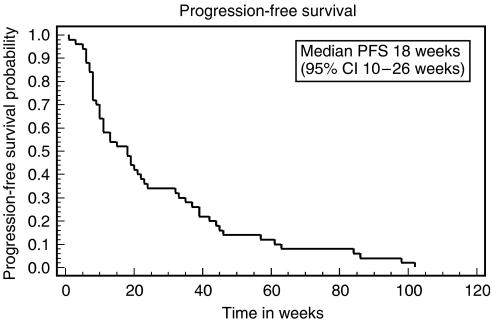
Kaplan–Meier plot of progression-free survival – all patients (ITT).

**Figure 2:**
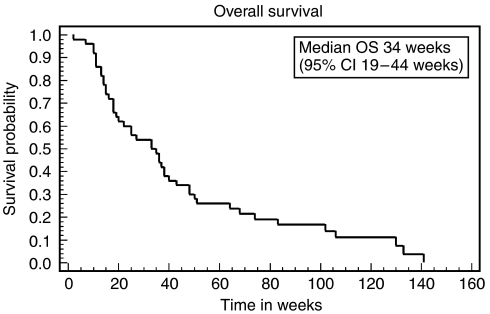
Kaplan–Meier plot of overall survival – all patients (ITT).

The response rate for evaluable patients was 30.5% (95% CI 19–44%). By ITT analysis the response rate was 28% (95% CI 15–40%). The median duration of response among the responders was 39 weeks (9 months). The ‘best outcome’ achieved in the nonresponders was 39% stable disease and 30% disease progression. Median progression-free survival for all patients (ITT) was 18 weeks (4.2 months) range 2–102+ weeks (95% CI 10–26 weeks) (see Figure 1). The median overall survival was 34 weeks (7.8 months) range 2–141 weeks (95% CI 19–44 weeks) (see Figure 2). The 12-month survival rate was 26% and the 24-month survival rate was 12%.

Subgroup analysis is depicted in Table 3. Whereas women had an overall better response rate than men, there was no difference in survival between the sexes. No significant median survival differences were observed with respect to sites of metastatic disease. There was an apparent trend towards greater response rate and survival with better performance status.

There was a trend towards greater response rates with increasing age. Patients aged less than 65 years had a response rate of 15% compared with 40% for patients aged 65 years or older. There was, however, similar overall survival for most patient age subgroupings, except for a small subgroup of patients aged less than 50 years with a median survival of 22 weeks.

## DISCUSSION

Fifteen years ago, the incidence of CUP was reported to be 5–10%. With improvements in diagnostic procedures, both in terms of accuracy and comfort, the current incidence of CUP is now reported as 2–5%. However, despite these substantial improvements in diagnostic procedures, a significant number of cancer patients will not have the primary site of their cancer discovered. Therefore, palliative chemotherapy protocols with a broad range of activity are still required for this clinical scenario. The challenge is to develop cytotoxic chemotherapy regimens that have a broad level of activity with minimal toxicity.

Historically, CUP regimens have often included fluoropyrimidines because of the predominance of occult GI primaries in this scenario. Because of the improvements in outcomes with chemotherapy for metastatic colorectal cancer and the relative ease of colonoscopy, colorectal cancer is nowadays specifically screened for in the setting of newly diagnosed CUP. With colorectal cancers being more routinely excluded in the diagnostic work-up, fluorouracil has become a less important agent in combination chemotherapy for CUP.

More recently, agents including taxanes, etoposide, gemcitabine and irinotecan have been used in combination with platinum compounds with apparent improved response rates and survival times. A 39% response rate with a 13-month median survival was achieved in a group of 77 CUP patients with a median age of 60 years who were treated with the carboplatin/paclitaxel combination (Briasoulis *et al*, 2000). The triplet of carboplatin/gemcitabine/paclitaxel followed by weekly paclitaxel for responders was used in 120 CUP patients with a median age of 59 years, and for these patients, a 25% overall response rate was achieved with a median survival of 9 months (Greco *et al*, 2002). In both of these studies, alopecia was almost universal and grade 3/4 myelosuppression was common, although CSFs were frequently used. Significant neuropathy was also common.

The GC combination used in our study could be reasonably used as first-line therapy in the setting of nonsmall-cell lung cancer, pancreatic/biliary cancer, bladder cancer, ovarian cancer and could be a valid second line combination for breast cancer. It has some activity in gastric cancer, but would certainly be considered an inferior treatment for colorectal cancer. The slightly lower dose of gemcitabine used in this study and the absence of taxane from the regimen has meant that toxicity was substantially reduced. In particular, there was almost no alopecia or neuropathy. Although CSFs were not used, the rate of significant myelosuppression was low.

The median age of the patients in this study was 69 years, about a decade older than the two previously described studies (Briasoulis *et al*, 2000, Greco *et al*, 2002). The response rate and overall survival achieved in our study is less than that demonstrated by Briasoulis *et al* (2000). It should be noted, however, that 21% of the patients entered in the Briasoulis study were women with peritoneal carcinomatosis who achieved a response rate of 81% (to paclitaxel/carboplatin) and it is possible that the inclusion of these patients may have substantially increased the overall response rate for the whole group. The response rate and overall survival in our study is more consistent with that of the study of Greco *et al* (2002), where there appears to have been tighter inclusion criteria.

Several studies have evaluated prognostic factors in CUP patients including sex, sites of disease, performance status and certain blood parameter variables. Some have suggested that male patients and presence of liver metastases were independent poor prognostic variables (Culine *et al*, 2002). However, in our study, while some of these parameters appeared to be associated with lower response rates, they did not appear to influence overall survival. The only variables that appeared to be associated with both lower response rate and lower overall survival were PS 2 and patients aged less than 50 years, although these subgroups constituted a small number of patients only.

Older patients in particular appeared to have a higher response rate with this regimen and median overall survival of this group was at least as good as the median survival for the whole group. Elderly women seemed to fare particularly well. Mammography was routine for all women entering the study and reasonable means to exclude likely ovarian or primary peritoneal carcinoma was routinely undertaken. For the four women in the trial who had peritoneal or mesenteric adenopathy as part of their disease, all had other sites of disease, including liver, lung or retroperitoneal adenopathy and all had tumour immunohistochemistry evaluation, which was more consistent with gastrointestinal cancer than gynaecological cancer. Two of these patients achieved a response. If occult breast cancer or ovarian cancer with variant histology was over-represented in this subgroup, then this may partly explain the better responses and outcomes. However, the results suggest that a pragmatic treatment approach of GC in such cases can be applied with reasonable efficacy. Additionally, the dosing and scheduling of this GC combination chosen was very tolerable in the elderly so that advanced age alone should not be a contraindication to treatment.

It is likely that genetic profiling of CUP in the future may not only vastly improve the likelihood of a primary site being eventually discovered, but may also dictate the appropriate treatment strategies regardless of possible primary sites. However, until such approaches are fully validated, regimens with a broad range of activity with good tolerability will be required to manage this common clinical entity. From a pragmatic viewpoint, when CUP is diagnosed in the absence of specific clinical situations that require tumour-specific treatment approaches, and if a colorectal primary can be reasonably excluded, the GC combination provides an active and very well-tolerated treatment regimen that is particularly applicable to the elderly population.

## Figures and Tables

**Table 1:** Patient characteristics (*N*=50)

**Characteristic**	**No. of patients**	**%**
*Age (years)*		
Median	69	
Range	41–83	
		
*Sex*		
Male	23	46
Female	27	54
		
*ECOG status*		
0	10	20
1	28	56
2	12	24
		
*Sites of disease*		
Liver	38	76
Lung	16	32
Retroperitoneal adenopathy	10	20
Peritoneum/mesenteric nodes	6	12
Bone	6	12
Pleura	5	10
		
*No. of cycles of chemotherapy*		
Median	4	
Range	1–9	
		

ECOG=Eastern Cooperative Oncology Group.

**Table 2:** Incidence of grade 3–4 toxicity

**Grade 3/4 toxicity**	**Incidence (%)**
Neutropenia	14 (including one death)
Anaemia	10
Thrombocytopenia	8
Thromboembolism	6

**Table 3:** Response and survival by sex, performance status and age criteria

**Characteristic**	**% Response**	**Median survival (weeks)**
Male	19	33
Female	40	36
		
PS 0	40	58
PS 1	28	33
PS 2	16	18
		
Age <65 years	15	30
Age ⩾65 years	40	38
